# The burden, admission, and outcome of COVID-19 in Africa: protocol for a systematic review and meta-analysis

**DOI:** 10.1080/22221751.2020.1775499

**Published:** 2020-06-15

**Authors:** Degena Bahrey Tadesse, Gebreamlak Gebremedhn Gebremeskel, Guesh Gebreayezgi Asefa, Mebrahtu Abay, Gebre Teklemariam Demoz

**Affiliations:** a Department of Adult Health Nursing, School of Nursing, Aksum University, Aksum, Ethiopia; b Department of Epidemiology, School of Public Health, Aksum University, Aksum, Ethiopia; c Department of Clinical Pharmacy, School of Pharmacy, Aksum University, Aksum, Ethiopia

**Keywords:** Admission, Africa, COVID-19, burden, outcome

## Abstract

*Background*: According to the World Health Organization (WHO), the outbreak of coronavirus disease in 2019 (COVID-19) has been declared as pandemic and public health emergency that infected more than 5 million people worldwide at the time of writing this protocol. Strong evidence for the burden, admission, and outcome of COVID-19 has not been published in Africa. Therefore, this protocol will be served as a guideline to conduct a systematic review and meta-analysis of the burden, admission, and outcome of COVID-19 in Africa. *Methods*: Published and unpublished studies on the burden, admission, and outcome of COVID-19 in Africa and written in any language will be included. Databases (PubMed / MEDLINE, Google Scholar, Google, EMBASE, Web of Science, Microsoft Academic, WHO COVID-19 database, Cochran Library, Africa Wide Knowledge, and Africa Index Medicus) from December 2019 to May 2020 will be searched. Two independent reviewers will select, screen, extract data, and assess the risk of bias. The proportion will be measured using a random-effects model. Subgroup analysis will be conducted to manage hetrogeinity. The presence of publication bias will be assessed using Egger's test and visual inspection of the funnel plots. This systematic and meta-analysis review protocol will be reported per the PRISMA-P guidelines. *Conclusion*: This systematic review and meta-analysis protocol will be expected to quantify the burden, admission, and outcome of COVID-19 in Africa. *Systematic review registration*: This protocol was submitted for registration with the International Prospective Register of Systematic Reviews (PROSPERO) in March 2020 and accepted with the registration number: CRD42020179321(https://www.crd.york.ac.uk/PROSPERO).

## Background

Coronavirus disease 2019 (abbreviated “COVID-19)” is an infectious respiratory illness caused by a novel coronavirus, first identified in Wuhan, China, in December 2019. It is large class of virus that have been relatively widespread all across the world. The virus has low pathogenicity and high transmissibility capacity [1]. The Chinese authorities in Wuhan City, the capital of the province of Hubei, China, first announced this at the end of December 2019 [2–5]. After originating in Wuhan China last December 2019, COVID-19 has spead to atleast 200 countries and regionos. The coronavirus disease 2019 (COVID-19) outbreak was declared a public health emergency of international concern by the World Health Organization (WHO) [6]. According to the WHO, the outbreak of coronavirus disease in 2019 (COVID-19) has been a pandemic that infected more than 5 million people at the time of writing this protocol and caused more than 324,000 deaths and 1.7 million recoveris worldwide within the six months duration [7]. Off the total cases 15% develop severe disease, including pneumonia, and 5% become critically ill with respiratory failure, septic shock and/or multi-organ failure [8]. In Africa, morbidity and mortality reached greater than 12,492 and 649 respectively [7].

Currently, COVID-19 affect 54 African countries with a total 103, 875 casese, 3,184 death,41,576 recoveries (with the highest cases in South Africa) and 64, 388 cases were in the WHO African region [9].

A modelling study in Africa, classifying African countries as having high risk with 13 top WHO highest pririty(Egypt, Algeria, and South Africa), moderate risk(Nigeria, Ethiopia, Morocco, Sudan, Angola, Tanzania, Ghana, and Kenya) and all other countries had low to moderate importation risk and low to moderate. The fatality rate of COVID in north African countries expected to be 11.02% [10, 11].

At the end of the pandemic, Africa will have some of the worst consequences of this COVID-19 pandemic. Generally speaking, African countries have poor health systems and this remains a source of concern, particularly in the event of an increase in outbreaks [12].

In WHO Africa region 83,000–190, 000 people could die of COVID-19 and 29 million to 44 million could get infected in the first year of the pandemic if coinainment measures fails. The research, which is based on prediction modelling, looks aat 47 countries in the WHO African Region with a total population of one million. There would be an estimated these 3.6–5.5 million COVID-19 hospitalization, of which 82,000–167,000 would be severe cases requiring oxygen, and 52,000–107,000 would be critical cases requiring bbreathing support [13].

There were different reports regarding the COVID-19 [10, 12, 14] but; there is no pooled results regarding the burden, admission and outcome of COVID-19 in Africa. Therefore, this study protocol will be guided to conduct a systematic review and meta-analysis of the burden, admission, and outcome of COVID-19 in Africa.

## Methods

### Protocol registration

This review is registered in PROSPERO International Prospective Register of Systematic reviews (CRD42020179321) (https://www.crd.york.ac.uk/PROSPERO) and reported according to Preferred Reporting Items for Systematic reviews and MetaAnalysis protocol (PRISMA-P) guidelines [15] (Table 1).

**Table 1.** PRISMA-P (Preferred Reporting Items for Systematic review and Meta-Analysis Protocols) 2015 checklist: recommended items to address in a systematic review protocol.

Section/topic	Item No	Checklist item	Information reported	Line number(s)
			Yes	No
Administrative Information				
Title:				
Identification	1a	Identify the report as a protocol of a systematic review		
Update	1b	If the protocol is for an update of a previous systematic review, identify as such		
Registration	2	If registered, provide the name of the registry (e.g. PROSPERO) and registration number in the Abstract		
Authors				
Contact	3a	Provide name, institutional affiliation, and e-mail address of all protocol authors; provide physical mailing address of corresponding author		
Contributions	3b	Describe contributions of protocol authors and identify the guarantor of the review		
Amendments	4	If the protocol represents an amendment of a previously completed or published protocol, identify as such and list changes; otherwise, state plan for documenting important protocol amendments		
Support				
Sources	5a	Indicate sources of financial or other support for the review		
Sponsor	5b	Provide name for the review funder and/or sponsor		
Role of sponsor/funder	5c	Describe roles of funder(s), sponsor(s), and/or institution(s), if any, in developing the protocol		
Introduction				
Rationale	6	Describe the rationale for the review in the context of what is already known		
Objectives	7	Provide an explicit statement of the question(s) the review will address with reference to participants, interventions, comparators, and outcomes (PICO)		
Methods				
Eligibility criteria	8	Specify the study characteristics (e.g. PICO, study design, setting, time frame) and report characteristics (e.g. years considered, language, publication status) to be used as criteria for eligibility for the review		
Information sources	9	Describe all intended information sources (e.g. electronic databases, contact with study authors, trial registers, or other grey literature sources) with planned dates of coverage		
Search strategy	10	Present draft of search strategy to be used for at least one electronic database, including planned limits, such that it could be repeated		
Study Records				
Data management	11a	Describe the mechanism(s) that will be used to manage records and data throughout the review		
Selection process	11b	State the process that will be used for selecting studies (e.g. two independent reviewers) through each phase of the review (i.e. screening, eligibility, and inclusion in meta-analysis)		
Data collection process	11c	Describe planned method of extracting data from reports (e.g. piloting forms, done independently, in duplicate), any processes for obtaining and confirming data from investigators		
Data items	12	List and define all variables for which data will be sought (e.g. PICO items, funding sources), any pre-planned data assumptions and simplifications		
Outcomes and prioritization	13	List and define all outcomes for which data will be sought, including prioritization of main and additional outcomes, with rationale		
Risk of bias in individual studies	14	Describe anticipated methods for assessing risk of bias of individual studies, including whether this will be done at the outcome or study level, or both; state how this information will be used in data synthesis		
*Data*	* *	* *	* *	* *
Synthesis	15a	Describe criteria under which study data will be quantitatively synthesized		
	15b	If data are appropriate for quantitative synthesis, describe planned summary measures, methods of handling data, and methods of combining data from studies, including any planned exploration of consistency (e.g. *I* ^2^, Kendall's tau)		
	15c	Describe any proposed additional analyses (e.g. sensitivity or subgroup analyses, meta-regression)		
	15d	If quantitative synthesis is not appropriate, describe the type of summary planned		
Meta-bias(es)	16	Specify any planned assessment of meta-bias(es) (e.g. publication bias across studies, selective reporting within studies)		
Confidence in cumulative evidence	17	Describe how the strength of the body of evidence will be assessed (e.g. GRADE)		

### Search strategy and data extraction

The search strategy has been applied using Online Databases (PubMed / MEDLINE, Google Scholar, Google, EMBASE,Web of Science, Microsoft Academic, WHO COVID-19 database, Cochran Library, Africa Wide Knowledge, and Africa Index Medicus) from December 2019 to May 2020 (Table 2). During the PROSPERO registration a total 10 articles were identified. The search terms which shall be used: “Wuhan coronavirus” OR “COVID-19” OR “novel coronavirus” OR “2019-nCoV” OR “coronavirus disease” OR “SARS-CoV-2” OR “SARS2” OR “severe acute respiratory syndrome coronavirus 2” OR “admission” OR “Burden” OR “Outcome”. Other searching terms will be used “mortality” OR “prevalence” OR “incidence” OR “cardiovascular complications” OR “renal complications” “hematological complications of COVID-19” OR “prevelence of asymptomatic, mild, moderate and severe cases” OR “admission” (“number admitted to specialized units or intensive care units” OR “outcome”) (“number of infected patients OR” “ number of recoveries” OR “case fatality rate” OR “ number of cured patients readmitted” OR “longterm complications”) such as chronic heart failure, cardiac arrhythmias and recurrent thromboembolic diseases.

**Table 2.** Searching strategy.

Serial number	Databases	Number of article found	Number of article included	Number of Excluded article	Reason for exclusion
1	PubMed	n=	n=	n=	
2	Google Scholar	n=	n=	n=	
3	Web of Science	n=	n=	n=	
4	Cochran Library	n=	n=	n=	
5	Africa Wide Knowledge	n=	n=	n=	
6	Africa Index Medicus	n=	n=	n=	
7	Microsoft Academic	n=	n=	n=	
8	WHO COVID-19 database	n=	n=	n=	
9	Unpublished (pre-prent, manuscript, thesis and report from WHO,CDC)	n=	n=	n=	

Searching results will be independently evaluated by two different reviewers. The literature search technique will be developed using the headings of the medical subject headings (Mesh), BOOLEAN (AND/OR) operator will be used.

The blinding will be maintained by using the Royyan that allows/ obligates each reviewer to work without knowing the other reviewer's choice. This review will be created using the metadata of the “COVID-19 Open Research Dataset” (https://pages.semanticscholar.org/coronavirus-research) (updated May 2020). We only uploaded the metadata (reference) file of 63k+ coronavirus and COVID-19 research articles with links to PubMed, Microsoft Academic and the WHO COVID-19 database of publications. We had to transform the metadata file (using https://github.com/rayyanqcri/CORD-19-importer) to make it compatible with Rayyan. You can export the data in this review and use it in Rayyan as usual for a systematic/literature review or to label the set for you downstream analytical task. Remember that you can work with multiple collaborators in a blinded or unblinded mode while chatting with them.

## Selection and data collection process

Data will be extracted using a standardized data extraction form. From the studies included, two reviewers (DBT and GTD)will independently extract data using the predefined standardized extraction form.. Full texts for the eligible titles and/or abstracts including those where there is uncertainty will be obtained for further assessment on whether to include in the study or not. Where necessary, authors will be contacted for additional information to confirm eligibility of studies. The agreement between review authors will be measured using Cohen's κ statistic. Disagreements will be resolved through discussion and when needed there will be arbitration by a third reviewer(MA). Reasons for excluding articles will be recorded.

Where there is missing information, the corresponding author of the study will be contacted to request the missing information. A maximum of three emails will be sent to the corresponding author to request for additional information before excluding the study. For studies appearing in more than one published article, we will consider the most recent, comprehensive, and with the largest sample size. For surveys appearing in one article with multiple surveys conducted at different time points, we shall treat each survey as a separate study. For multi-national studies, data will be separated to show the estimate at the country level.

Data extracted comprised information about the month of publication, country, and design of the study, admission rate, burden, outcome, diagnostic criteria, comorbidity, COVID-19, mean age,ethinicity, sex (male proportion), signs and symptoms, complications, prevalence and/or incidence, and risk factors.

## Inclusion and exclusion criteria

Studies presented as original articles, studies that assessed burden, admission, and outcome from COVID-19 will be included.

*Types of studies*: Observational studies (including cross-sectional, case–control, and cohort) and randomized controlled trial will be included. In the case of duplicate reports, the most comprehensive and up-to-date version will be taken into account.

*Participants*: All Patients who are African residence and will be diagnosed as having COVID-19.

*Intervention(s)/exposure(s)*: Demographic, clinical, laboratory, management, and outcome data will be reviewed.

*Outcome*: Epidemiological data, admission pattern, mortality, and clinical outcomes of COVID-19. Establishing the clinical and epidemiological features, outcomes of COVID-19.

*Settings*: Hospital-based studies.

*Publication date*: December 2019 to May 2020.

*Language*: all published and unpublished papers (thesis,manuscript, pre-print pending to be published, report from WHO, Comminicable Diseases Control, United Nation and health authorities in different African countries) without restriction of language will include in our review.

*Method of diagnosis*: No restriction on methods of diagnosis but we will conducted subgroup analysis based on diagnostic tools. WHO interim guidance and /or any WHO recommended diagnostic criteria will be considered[16] (Table 3).

**Table 3.** Laboratory testing for coronavirus disease (COVID-19) in suspected human cases: interim guidance.

Test	Type of sample	Timing
Nucleic acid amplification tests (NAAT)	**Lower respiratory tract** Sputum Aspirate Aavage **Upper respiratory tract** Nasopharyngeal and Oropharyngeal swabs Nasopharyngeal wash/nasopharyngeal aspirate.	Collect on presentation. Possibly repeated sampling to monitor clearance. Further research needed to determine effectiveness and reliability of repeated sampling.
Serology	Serum for serological testing once validated and available.	Paired samples are necessary for confirmation with the initial sample collected in the first week of illness and the second ideally collected 2–4 weeks later (optimal timing for convalescent sample needs to be established).

*Exclusion criteria*: Studies that did not explain the criteria for the level COVID-19 outcome; studies that didn't state the number of patients with COVID-19 will be excluded. Studies not performed in humans, qualitative studies, studies that lack relevant data needed to compute the burden, admission, and outcome will be excluded. Experimental studies, letters, reviews, commentaries, editorials, case reports, or case series will be not included.

## Quality assessment and risk of bias in individual studies

To assess the risk of bias and quality of studies included in this review, a tool developed by Hoy et al. for prevalence studies will be used [17] The tool contains 11 items; items 1–4 assess the external validity, 5–10 assess the internal validity, and item 11 is a summary of the overall risk by the reviewer based on the responses of the above 10 items which are scored 1 if yes and 0 if no. Studies will be classified as having a low (> 8), moderate or high (≤ 5) risk of bias. Additional file 1 shows this in more detail regarding the checklist of bias measurement on the observational study. For RCTs, we will use SPIRIT 2013 Checklist [18]. Additional file 2 shows this in more detail regarding the checklist of SPIRIT.

## Data management

Based on the inclusion and exclusion criteria, a tool has been developed a priori to guide the screening and selection process. The tool will be piloted and revised before data extraction begins. The search results will be uploaded to EndNote software first to remove duplicates.

## Data items

Data on general information, authors, month, country, and region, type of publication, study characteristics (study design, setting, sample size, response rate, mean or median age, or age range), outcome, burden, and admission rate will be extracted.

## Outcomes and prioritization

The primary outcome is the burden, admission, and outcome of COVID-19 in Africa.

## Data synthesis, analysis and presentation

Data will be analyzed using the R software; V.3.5.3. Data will be summarized using ranges, means ±SDs, and frequencies (percentages) where appropriate. All analyses will be performed using a “metaprop” routine using R version 3.5.3 for Windows [19]. Results will be reported as proportions with corresponding 95% confidence intervals (CIs). Forest plots will be drawn to visualize the combined burden, admission, and outcome of COVID-19 and the extent of statistical heterogeneity between studies. Statistical heterogeneity will be assessed using theχ2 test on Cochrane's Q statistic, 20 and quantified by calculating the I^2^ statistic (with values of 25%, 50%, and 75% is representative of the low, medium, and high heterogeneity, respectively) [20]. There will be a clinical heterogeneity between studies included in this study. We will used a random-effects meta-analysis to estimate the overall pooled burden, admission, and outcome of COVID-19 in Africa. The presence of publication bias will be assessed using Egger's test and funnel plots [21]. *P*-value < 0.10 on the Egger's test will be considered statistically significant for publication bias. Moreover, other relevant findings will be summarized in a narrative format.

Crude numerators and denominators from the individual studies will be used to recalculate the study-specific prevalence/burden, admission, and outcome of COVID-19. Prevalence estimates will be summarized by African geographic regions.

A meta-analysis will be performed on variables that are similar across the included studies.

Subgroup analyses will be performed based on the countries where the study conducted, diagonostic method they used, based on their ethinic background(African origin and non African ethinic origin): The definitions of the comorbidities of interest will be collected, and those with the same definitions will be analyzed together. Inter-rater agreements between the researchers involved in study selection and those involved in the identification of risk of bias will be assessed using *κ* Cohen's coefficient.

## Discussion

**Figure 1.**
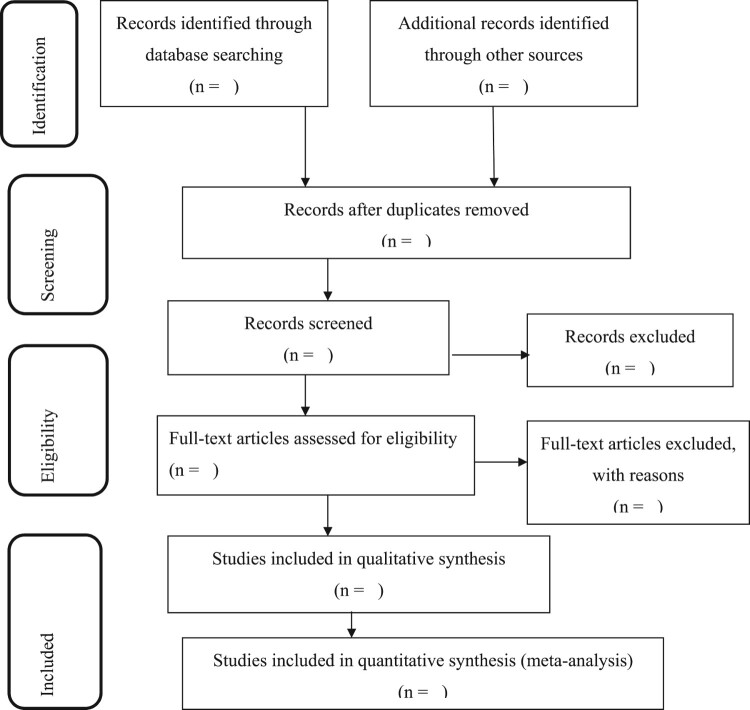
Flow chart diagram will be showed the selection of articles for systemic review and meta-analysis of the burden, admission, and outcome of COVID-19 in Africa.

This review will be published per the PRISMA-P guidelines [15]. The PRISMA flow diagram will be used to record the different phases of the review process [15] (Figure 1).

The summary will be used to display the data on the distribution of COVID-19 by variables of interest such as gender, residence, setting, and person-level characteristics. Funnel plots will be used to visualize publication bias of the included studies. Forest plots will be used to estimates the burden, admission rate, and outcome of COVID-19 for the included studies as an overall pooled estimate for Africa. Results from this review will inform healthcare providers on the burden, admission rate, and outcome of COVID -19, hence providing evidence will bring the required changes needed in clinical practice and will support healthcare services in line with patients’ needs. Findings from this review will be shared in conferences, peer-review journals, and social media platforms.

*Conclusion*: This systematic review and meta-analysis will be expected to quantify the burden, admission, and outcome of COVID-19 in Africa to guide policies and interventions.

## Supplementary Material

Supplemental Material

## Data Availability

The datasets used and/or analyses during the study will be presented within the manuscript and available from the corresponding author on reasonable request.

## Abbreviations

Abbreviations: PRISMA-P: Preferred Reporting Items for Systematic reviews and Meta-Analysis protocol; WHO: World Health Organization; COVID-19: Coronavirus Disease in 2019
