# Supplementary material for: The burden, admission, and outcome of COVID-19 in Africa: protocol for a systematic review and meta-analysis
Source: Emerg Microbes Infect. 2020 Jun 15;9(1):1372–8. doi: 10.1080/22221751.2020.1775499 (PMC7473325; doi:10.1080/22221751.2020.1775499)
Supplement: Supplemental Material [file TEMI_A_1775499_SM2588.zip › 1775499/3 Hoy Checklist -additional file2.docx]

Table. Quality assessment checklist for prevalence studies (adapted from Hoy et al

| S/N | Risk of bias items | Risk of bias levels | Points scored |
| --- | --- | --- | --- |
| 1 | Was the study’s target population a close representation of the national population in relation to relevant variables, e.g. age, sex, occupation? | Yes (LOW RISK): The study’s target population was a close representation of the national population. | 0 |
|  |  | No (HIGH RISK): The study’s target population was clearly NOT representative of the national population. | 1 |
| 2 | Was the sampling frame a true or close representation of the target population? | Yes (LOW RISK): The sampling frame was a true or close representation of the target population. | 0 |
|  |  | No (HIGH RISK): The sampling frame was NOT a true or close representation of the target population. | 1 |
| 3 | Was some form of random selection used to select the sample, OR, was a census undertaken? | Yes (LOW RISK): A census was undertaken, OR, some form of random selection was used to select the sample (e.g. simple random sampling, stratified random sampling, cluster sampling, systematic sampling). | 0 |
|  |  | No (HIGH RISK): A census was NOT undertaken, AND some form of random selection was NOT used to select the sample. | 1 |
| 4 | Was the likelihood of non-response bias minimal? | Yes (LOW RISK): The response rate for the study was ≥75%, OR, an analysis was performed that showed no significant difference in relevant demographic characteristics between responders and non- responders | 0 |
|  |  | No (HIGH RISK): The response rate was <75%, and if any analysis comparing responders and non-responders was done, it showed a significant difference in relevant demographic characteristics between responders and non-responders | 1 |
| 5 | Were data collected directly from the subjects (as opposed to a proxy)? | Yes (LOW RISK): All data were collected directly from the subjects. | 0 |
|  |  | No (HIGH RISK): In some instances, data were collected from a proxy. | 1 |
| 6 | Was an acceptable case definition used in the study? | Yes (LOW RISK): An acceptable case definition was used | 0 |
|  |  | No (HIGH RISK): An acceptable case definition was NOT used | 1 |
| 7 | Was the study instrument that  measured the parameter of interest  (e.g. prevalence of low back pain)  shown to have reliability and validity (if necessary)? | Yes (LOW RISK): The study instrument had been shown to have reliability and validity (if this was necessary), e.g. test-re- test, piloting, validation in a previous study, etc. | 0 |
|  |  | No (HIGH RISK): The study instrument had NOT been shown to have reliability or validity (if this was necessary) | 1 |
| 8 | Was the same mode of data collection used for all subjects? | Yes (LOW RISK): The same mode of data collection was used for all subjects. | 0 |
|  |  | No (HIGH RISK): The same mode of data collection was NOT used f or all subjects. | 1 |
| 9 | Were the numerator(s) and  denominator(s) for the parameter of interest appropriate | Yes (LOW RISK): The paper presented appropriate numerator(s) AND denominator(s) for the parameter of interest (e.g. the prevalence of low back pain). | 0 |
|  |  | No (HIGH RISK): The paper did present numerator(s) AND denominator(s) for the parameter of interest but one or more of these were inappropriate. | 1 |
| 10 | Summary on the overall risk of study  bias | LOW RISK | 0-3 |
|  |  | MODERATE RISK | 4-6 |
|  |  | HIGH RISK | 7-9 |
